# Large lungs may predict increased air trapping in navy divers

**DOI:** 10.14814/phy2.15153

**Published:** 2022-02-25

**Authors:** Tomi Wuorimaa, Jari Haukka, Janne Tikkinen, Kai Parkkola, Päivi Piirilä

**Affiliations:** ^1^ Diving Medical Centre Centre for Military Medicine Upinniemi Finland; ^2^ Department of Clinical Physiology and Nuclear Medicine of HUS Medical Diagnostic Center Laboratory of Clinical Physiology Helsinki University Hospitals Helsinki Finland; ^3^ Clinicum/Department of Public Health University of Helsinki Helsinki Finland; ^4^ Faculty of Medicine and Health Technology Tampere University Tampere Finland; ^5^ National Defence University Helsinki Finland

**Keywords:** air trapping, diving, large lungs, obstruction, reserve volume

## Abstract

Navy divers tend to have large lungs and low expiratory flow rates in the terminal portion of a spirogram. We examined Finnish Navy divers for the presence of air trapping, airway obstruction, and functional airway compression, and their association with lung volumes. Divers (*n* = 57) and non‐diving men (*n* = 10) underwent a variety of pulmonary function tests. The amount of trapped air was calculated as the subtraction of the total lung capacity (TLC) measured in a single‐breath helium dilution test from the TLC in body plethysmography (TLCb). Mean vital capacity (VC) was 6.4 L in the divers versus 5.8 L in the controls (*p* = 0.006) and TLCb 8.9 L in the divers versus 8.1 L in the controls (*p* = 0.002). No difference existed between them in the amount of trapped air. However, we found break points in a linear regression model (Davies test) between trapped air and several pulmonary parameters. Those individuals above the break points had lower ratio of forced expiratory volume in first second to forced vital capacity, lower resistance of airways, and higher reactance than those below the break points. In conclusion, navy divers had larger lungs than controls. Large lung volumes (VC >7.31 L or >122% of predicted value) were associated with air trapping. Furthermore, large volumes of air trapping (>1.1 L) were associated with increased residual volume (RV) and RV/TLCb. Despite no concurrent obstruction, functional airway compression, or reduced diffusing capacity, this slowly ventilated trapped air might remain disadvantageous for divers.

## Graphical abstract

Large lung volumes (VC >7.31 L or >122% of predicted value) were associated with air trapping. Furthermore, large volumes of air trapping (>1.1 L) were associated with increased residual volume (RV) and RV/TLCb. Despite no concurrent obstruction, functional airway compression, nor reduced diffusing capacity, this slowly ventilated trapped air might remain disadvantageous for divers.

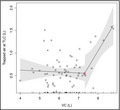

## 1 BACKGROUND

Many professional and military divers have large lungs and signs of bronchial obstruction without actual pulmonary disease (Adir et al., 2005; Crosbie et al., 1979; Fitzpatrick & Conkin, 2003; Konarski et al., 2013; Reuter et al., 1999; Skogstad et al., 2000). Many divers have large lungs even before they start diving, and during the first years of a diving career, vital capacity (VC) and forced vital capacity (FVC) tend to increase even further (Brubakk & Neuman, 2003; Crosbie et al., 1979; Fitzpatrick & Conkin, 2003; Skogstad et al., 2000). Long‐term diving exposure seems to also promote small airway obstruction, indicated by reduced maximal expiratory flow rates, at least at the terminal part of the flow–volume curve, and possible a small decrease in forced expiratory volume in the first second (FEV1) (Brubakk & Neuman, 2003; Davey et al., 1984; Skogstad & Skare, 2008; Skogstad et al., 2000, 2002; Tetzlaff et al., 1998; Thorsen et al., 1990). However, according to a recent study from New Zealand long‐term exposure of occupational diving caused only small changes in spirometric measurements, and the changes were largely explained by increasing age (Sames et al., 2018).

The obstructive changes in small airways in divers are probably not clinically significant. However, it is widely accepted that marked local airway obstruction can cause air trapping in the lungs and increase the risk of pulmonary barotrauma (PBT) during ascent. Several reports and studies with divers have concluded that reduced end‐expiratory flow or small, pre‐existing lung cysts or blebs may increase the risk of PBT (Benton et al., 1999; Bove, 1997; Hartge & Bennet, 2015; Reuter et al., 1997; Tetzlaff et al., 1997), but no association existed between a low FEV1/VC or FEV1/FVC ratio and PBT (Benton et al., 1999; Reuter et al., 1999). In contrast to this, a low VC and FVC are associated with the incidence of PBT (Benton et al., 1999; Brooks et al.). It has been speculated that reduced VC is associated with a reduced compliance of the lungs, thereby predisposing them to the rupture of alveoli during free ascent (Benton et al.).

Enlarged lung volumes may also create circumstances that predispose divers to lung injury. A small report previously suggested the common factor among Dutch military divers suffering PBT was a large lung volume: an FVC value above 120% of the predicted value (Hulst et al., 2011). These divers also showed air trapping, large bulla, and multiple blebs, documented by computerized chest tomography (HRCT) (Hulst et al., 2011; Mets et al., 2012). However, a high prevalence of lobular air trapping observed in HRCT in Dutch military divers weakens the causative association between air trapping and PBT.

Several physiological mechanisms can interfere with pulmonary function or contribute to the risk of pulmonary damage as well. Forced exhalation leads to physiological flow limitation, which is called *functional airway compression*. In this phenomenon, bronchioles partly collapse during forced exhalation, impeding outgoing airflow from alveoli. At the end‐expiratory phase, this leads to the full collapse of bronchioles, causing functional airway compression and air trapping in peripheral airways (Pellegrino et al., 1994; Sood et al., 2019). Trapped air is a potential risk during ascent when diving, particularly when occurring simultaneously with an airway obstruction caused by other mechanisms (Dahlback & Lundgren, 1972). Although functional flow limitation and airway compression phenomena are physiological, they can be aggravated by pathological conditions or pulmonary diseases (Piirilä et al., 2014), by increased ventilation during physical stress, and by the increased density of inhaled gas with increased diving depth (Mitchell et al., 2007).

Although there are no established reference values for normal amount of trapped air, some scientific evidence do exist. In normal healthy subjects, the mean trapped air has been ranging between 0.2 and 0.4 L (Schaaning & Gulsvik, 1973; Sovijärvi et al., 1980). Among patients with chronic obstructive pulmonary disease and emphysema, mean volumes of trapped air have been reported ranging between 0.4 and 1.4 L. Even 3–4 L trapped air volumes have been found in some severe cases.

The aim of this study was to characterize the lung function profiles of Finnish Navy divers to see whether they show features of pathological air trapping or functional airway compression and see how the possible air trapping is associated with lung volumes.

## 2 METHODS

In the Finnish Navy, divers are routinely examined as a part of occupational health care. The examinations include a variety of lung function and cardiopulmonary fitness tests. Normal lung function is required for medical fitness to dive. We utilized the data collected from all examinations performed between 2010 and 2015. The study protocol was approved by the Logistic Department of the Defence Command Finland (AJ12177/11.6.2013 and AR1737/1073/12.04.01/2020) and the study adhered to the Declaration of Helsinki. According to the Ethics Committee of Helsinki University Central Hospital, no approval by the committee is needed because the study is performed retrospectively by investigating data collected during routine occupational health assessment.

### 2.1 Divers and non‐diving individuals

We characterized the pulmonary function profiles of 57 (*N* = 57) healthy professional Finnish Navy divers and 10 (*N* = 10) healthy non‐diving individuals (see Table 1). All the divers and non‐diving individuals were male and mostly non‐smokers. The divers were regular divers utilizing air or nitrox as breathing gas, and their diving depth range was 1–50 m; no saturation divers were included.

**TABLE 1:** Basic characteristics of navy divers and controls (mean and SD)

	Navy divers	Controls	*p*‐value
	(*N* = 57)	(*N *= 10)	
Age (years)	34.0 (9.86)	35.8 (4.54)	0.048
Height (cm)	180.8 (5.09)	177.9 (4.84)	0.071
Weight (kg)	83.0 (7.74)	76.8 (7.93)	0.007*
Body mass index (BMI)	25.7 (2.17)	24.2 (1.68)	0.387
Smoking	5.3%	0%	

*p*‐value for Wilcoxon rank‐sum test,

* *p* ≤ 0.05.

### 2.2 Pulmonary function tests

All pulmonary function tests were performed in the Laboratory of Clinical Physiology in the University Central Hospital of Helsinki. Spirometry was measured with a Medikro SpiroStar spirometer (Medikro Kuopio, Finland) using Finnish reference values (Viljanen, 1982) and according to American Thoracic Society (ATS) guidelines (Miller et al., 2005). For divers with an obstruction, in spirometry we performed a bronchodilation test using a salbutamol aerosol (0.4 mg) and measured post‐bronchodilator spirometry 15 min after the dose.

Single‐breath diffusing capacity for carbon monoxide and single‐breath helium (He) dilution tests were measured according to ATS and European Respiratory Society (ERS) guidelines (Macintyre et al., 2005) and the mean value of two measurements was the result. The results were corrected for actual hemoglobin concentration. We reported total diffusing capacity (DLCOc), specific diffusing capacity (DLCOc/VA), total lung capacity (TLC) measured by a single‐breath helium dilution test (TLC‐He), and residual volume (RV) measured by a single‐breath helium dilution test (RV‐He).

The plethysmographic measurements were first performed in conventional constant volume mode (MasterScreen Body Version 4.3, Würzburg, Germany) followed by flow plethysmography, as reported earlier (Piirilä et al., 2014). The atmospheric pressure was measured using a Vaisala device (Vaisala, Finland). Flow–volume calibration according to the three‐flow protocol of the device was performed with a Jaeger 3L calibration syringe. If the deviation of the registered volume was within ±3.5% for all flows, the calibration was regarded as successful. Box verification/calibration included two procedures. The time constant of the box was verified to lie within the range of 4–7 s. Box shift volume was calibrated following the recommendations of the manufacturer.

For flow plethysmography, corresponding calibration methods were applied. To allow flow–volume calibration of the spirometer connected to the box chamber, the box was set to the flow plethysmographic (transmural) mode by removing the closure on the box wall hole and closing the door of the box. The calibration pump strokes were directed through the box chamber pneumotachograph. A volume calibration was accepted if the stroke volumes did not exceed the ±3.5% range.

All investigations were first performed in the conventional constant volume mode. The measurements of specific resistance breathing loops and functional residual capacity (FRCpleth) were performed with a panting frequency of 0.5 Hz. The four‐second shutter maneuver (FRCpleth) was linked to a maximal spirometric slow VC maneuver for the determination of residual volume measured by body plethysmography (RVb) and total lung capacity measured by body plethysmography (TLCb). The system automatically derived total specific conductance (Sgeff) from the breathing loops and determined total respiratory resistance (Rtot).

In the flow plethysmographic mode, the uncompressed flow–volume curve at the chest wall and the compressed flow–volume loops at the mouth were measured simultaneously. The differences between compression free or thoracic flows and corresponding compressed mouth flows were regarded as rough estimates of the degree of gas compression at levels of peak flow (PEF) and maximal expiratory flows at 75%, 50%, and 25% of the remaining FVC (MEF75, MEF50, and MEF25, respectively). The volume reference was the same in both thoracic and mouth flow measurements, that is, in the FVC.

The amount of trapped air was calculated as the subtraction of TLC‐He from TLCb (Skogstad et al., 2000).

The determination of the single‐breath nitrogen test and closing volume (CV) was performed according to the method of Buist and Ross, and their reference values were also used (Buist & Ross, 1973). Impulse oscillometry was performed according to the method of Vogel and Smidt, and their reference values were used (Vogel & Smidt, 1994).

### 2.3 Statistics

We used the Wilcoxon rank‐sum test to compare the lung function results of the divers with those of the controls. We first modeled the association between trapped air as a predictor and other measurements as outcomes with a linear regression model. Next, we used the Davies test to check if there was a break point in the linear regression (Davies, 1987, 2002). If a break point was detected, data were modeled with a regression model for the break point (Muggeo, 2003, 2008) and two slopes are reported. We pooled the data of the divers and controls for regression analysis. *P*‐values are given merely for descriptive purposes in descriptive tables. In Table 4 also 95% confidence intervals are given and *p*‐values are reported as additional information. To control multiple comparisons, we calculated corrected q‐values and false discovery rates (Storey et al., 2004).

## 3 RESULTS

### 3.1 Spirometry and TLC

The navy divers had significantly larger lung volumes (in terms of VC, FVC, TLC‐He, and TLCb) than the healthy controls (see Table 2). Despite the fact that seven divers had a mild obstruction (FEV1/FVC%: 78%–87%; MEF50%: 35%–61%) and four divers had a moderate obstruction (FEV1/FVC%: 62%–77%), the FEV1 response in a bronchodilation test remained under 12% in all the divers.

**TABLE 2:** Characterization of lung parameters (mean and SD, percentage of reference value and SD)

	Navy divers	Controls	*p*‐value 1	*p*‐value 2
VC (L/%)	6.36 (0.74)/110 (10.96)	5.79 (1.02)/101 (12.08)	0.006*	0.008*
FVC (L/%)	6.31 (0.79)/112 (11.57)	5.75 (0.91)/103 (10.55)	0.009*	0.012*
FEV1 (L/%)	4.85 (0.70)/104 (11.58)	4.50 (0.38)/99 (6.46)	0.080	0.089
FEV1/FVC (%/%)	0.77 (0.07)/93 (7.81)	0.79 (0.06)/96 (7.02)	0.346	0.175
FEV1/VC (%/%)	0.76 (0.08)/94 (8.95)	0.79 (0.07)/98 (8.18)	0.234	0.093
PEF (L/s, %)	10.79 (1.62)/99 (14.69)	10.38 (1.41)/97 (11.99)	0.519	0.819
MEF50 (L/s, %)	5.10 (1.38)/85 (21.39)	5.07 (0.99)/88 (20.29)	0.833	0.833
MEF25 (L/s, %)	1.94 (0.84)/83 (27.03)	1.87 (0.49)/90 (30.91)	0.860	0.714
MMEF (L/s)	4.3 (1.21)	4.19 (0.76)	0.785	
TLC‐Helium dilution (L/%)	8.30 (0.85)/107 (10.38)	7.50 (1.15)/96 (10.45)	0.002*	0.002*
TLC‐bodypleth (L/%)	8.96 (0.99)/111 (11.99)	8.15 (1.51)/101 (13.46)	0.002*	0.004*
ERV‐bodypleth (L/%)	1.95 (0.59)/117 (33.92)	2.02 (0.72)/112 (35.38)	0.338	0.100
RV‐bodypleth (L/%)	2.33 (0.49)/101 (21.12)	2.14 (0.47)/90 (15.93)	0.109	0.086
RV/TLC‐bodypleth (%/%)	25.60 (4.78)/89 (15.16)	26.15 (2.48)/89 (8.12)	0.711	0.993
ITGV (FRC bodypleth) (L/%)	4.25 (0.61)/105 (15.62)	4.16 (1.17)/98 (22.22)	0.086	0.033
Rtot (kPa*s/%)	0.19 (0.05)/154 (34.64)	0.17 (0.05)/144 (37.08)	0.345	0.521
Sgeff (1/(kPa*s/%)	1.36 (0.36)/70 (18.66)	1.64 (0.56)/84 (28.26)	0.130	0.107
DLCOc (mmol/(min*kPa)/%)	11.60 (1.45)/104 (11.94)	10.56 (1.52)/96 (11.90)	0.050*	0.054
DLCOc/VA (mmol/(min*kPa*L)/%)	1.58 (0.18)/98 (10.86)	1.58 (0.19)/98 (11.61)	0.867	0.986
N2 slope (%)	2.17 (1.43)	1.71 (0.99)	0.431	0.336
CV/VC (%)	80.3 (41.6)	90.1 (21.4)	0.050*	
R5 (%)	93.8 (23.9)	90.4 (17.2)	0.928	
R20 (%)	96.5 (19.9)	90.5 (19.7)	0.335	
X5 (%)	−136.6 (1183.8)	−625.1 (439.8)	0.040*	
PEF difference^*^	0.03 (0.09)	0.01 (0.01)	0.734	
MEF75 difference^*^	1.75 (1.07)	1.39 (1.34)	0.167	
MEF50 difference^*^	1.73 (1.04)	1.59 (1.06)	0.641	
MEF25 difference^*^	0.56 (0.48)	0.52 (0.33)	0.902	

Note

*p*‐value 1 is the *p*‐value from the comparison of the measured values, *p*‐value 2 from the comparison of the values percent of predicted values, the spirometric values, and DLCOc calculated according to height and age, the body plethysmographic values, and DLCOc/VA according to age, height, and weight (Viljanen, 1982).

*p*‐value for Wilcoxon rank‐sum test,

^a^ PEF difference, MEF75 difference, MEF50 difference, and MEF25 difference represent gas compression (flow in transmural—flow in mouth) during forced expiration (L/s).

* *p* ≤ 0.05.

### 3.2 Other lung function parameters

No differences existed in plethysmographic parameters between the divers and controls (see Table 2). DLCOc was higher in the divers than in the controls (11.6 vs. 10.6; *p* = 0.05), but when adjusted with lung volumes, no difference remained in DLCOc/VA. In nitrography, the ratio between CV and VC and its predicted values (CV/VC and CV/VC%) tended to be lower in the divers than in the controls. In impulse oscillometry, the reactance percentage of the predicted value (X5%) was significantly higher in the divers than in the controls (*p* = 0.04). Otherwise, parameters in oscillometry were similar between the two groups. We found no difference in gas compression (as measured PEF‐MEF75, MEF50, and MEF25 difference in flow plethysmography) between the two groups, indicating a similar pattern in the functional airway compression of the divers and healthy controls (see Table 2).

### 3.3 Trapped air

**FIGURE 1:**
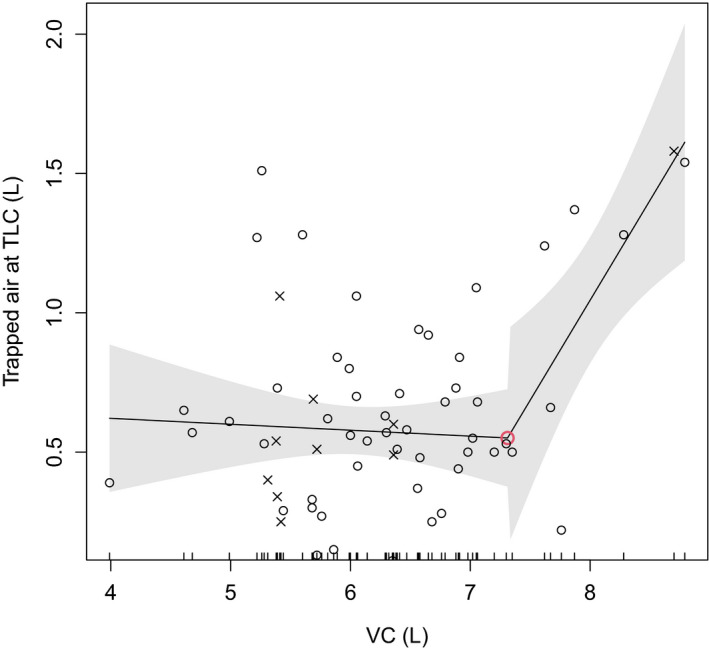
Vital capacity (VC, volume in liters) and trapped air (volume liters) modeled using change point regression. Navy divers are marked with ○ and control individuals with ×. The lines represent linear regression and the ○ represents a break point in the regression. The gray area depicts 95% confidence interval

**FIGURE 2:**
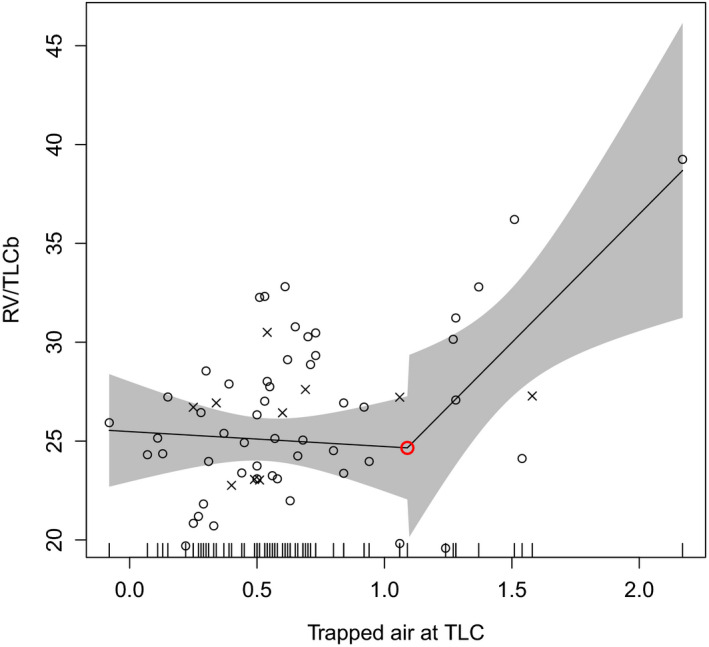
Trapped air (volume liters) and the function of residual volume (RV/TLCb) modeled using change point regression. Navy divers are marked with ○ and control individuals with ×. The lines represent linear regression and the ○ represents a break point in the regression. The gray area depicts 95% confidence interval

The volume of trapped air at TLC level was 0.66 L in the divers and 0.65 L in the controls, ranging from 0.07 to 1.58 L (see Table 3). We analyzed the relation of this trapped air to all other pulmonary functions. We pooled the data from divers and controls for the regression analysis. In a linear regression model, we found a significant (*p* < 0.03) correlation between trapped air and VC, VC%, FVC, FVC%, TLCb, TLCb%, TLC‐He, RV, RV%, RV/TLC, RV%/TLC, N2, N2%, and DLCO/VA. We found statistically significant (*p* < 0.01) break points between trapped air and VC, VC%, RV, RV%, RV/TLC, RV%/TLC, expiratory reserve volume (ERV), and ERV% in a regression with the Davies test (see Table 4). The relation of VC to trapped air was almost stable until the volume of about 7.31 L or 122% of the predicted value of VC; after that, the volume of trapped air started to increase (see Figure 1; break point 2, Table 4). As the volume of trapped air reached a break point of about 1.1 L in regression, then RV and RV/TLCb increased (see Figure 2; break point 5, Table 4) and at a volume of 1.6 L, ERV decreased (see Table 4). In other words, the relation of RV and RV/TLC to trapped air was almost stable until the volume of 1.1 L of trapped air, and then both RV and RV/TLC started to increase and later, with at a larger volume, ERV also started to decrease.

**TABLE 3:** Volume (liters) of air trapping at the level of total lung capacity (TLC)

	Navy divers Mean (SD) Median Range	Controls Mean (SD) Median Range	*p*‐value
Trapped air at TLC (L)	0.66 (0.42) 0.575 0.07–1.54	0.65 (0.40) 0.521 0.25–1.58	NS

*p*‐value for Wilcoxon rank‐sum test.

**TABLE 4:** Correlation between trapped air (volume in liters) and vital capacity (VC, volume in liters), capacity residual volume (RVb, volume in liters), RV/TLCb, expiratory reserve volume (ERV, volume in liters), and their percentage in predicted value (%) using change point regression. The break point represents the value where significant change in regression occurred with the Davies test. The slopes are for the lines in linear regression before and after a break point

	Parameter	Break point	SED and 95% CI	*p*‐value	Slopes	SED and CI95%
1. Trapped air versus VC	VC	7.31 L	0.30 (6.74–7.88)	0.0008	−0.021	0.061 (−0.14–0.09)
					0.718	0.23 (0.27–1.17)
2. Trapped air versus VC%	VC%	122%	4.30 (114–130)	0.001	0.002	0.01 (−0.13–0.01)
					0.055	0.02 (0.01–0.10)
3. Trapped air versus RVb	RVb	2.31 L	0.11 (2.10–2.52)	0.0008	−0.162	0.21 (−0.59–0.26)
					0.919	0.14 (0.65–1.19)
4. Trapped air versus RVb%	RVb%	149%	6.99 (135–163)	0.0002	0.008	0.003 (0.002–0.013)
					0.043	0.015 (0.013–0.073)
5. RV/TLCb versus trapped air	Trapped air	1.09 L	0.19 (0.71–1.47)	0.025	−0.754	2.14 (−5.04–3.53)
					12.995	4.96 (3.08–22.91)
6. RV/TLCb% versus trapped air	Trapped air	1.09 L	0.140 (0.82–1.36)	0.0005	−6.870	6.46 (−19.78–6.04)
					50.841	14.96 (20.96–80.73)
7. ERV versus trapped air	Trapped air	1.58 L	0.09 (1.40–1.76)	0.014	0.131	0.21 (−0.29–0.56)
					−4.745	1.68 (−8.11–−1.38)
8. ERV% versus trapped air	Trapped air	1.69 L	0.08 (1.54–1.84)	0.012	3.968	16.33 (−28.67–36.61)
					−236.34	70.83 (−377.89–−94.79)

In the Davies test, air trapping did not correlate with other indices of pulmonary pathology (parameters representing obstruction, functional airway compression, diffusion capacity, nitrography, or oscillometry).

**FIGURE 3:**
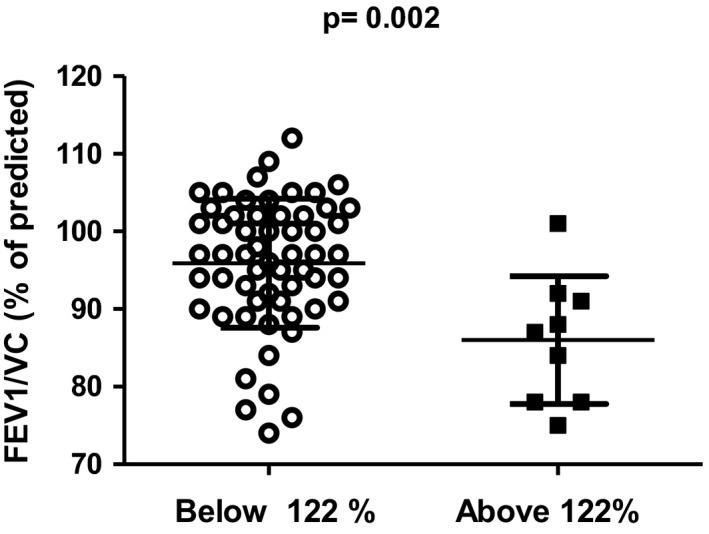
FEV1/FVC % of predicted below and above break point 2. Mean and standard deviation are given

**FIGURE 4:**
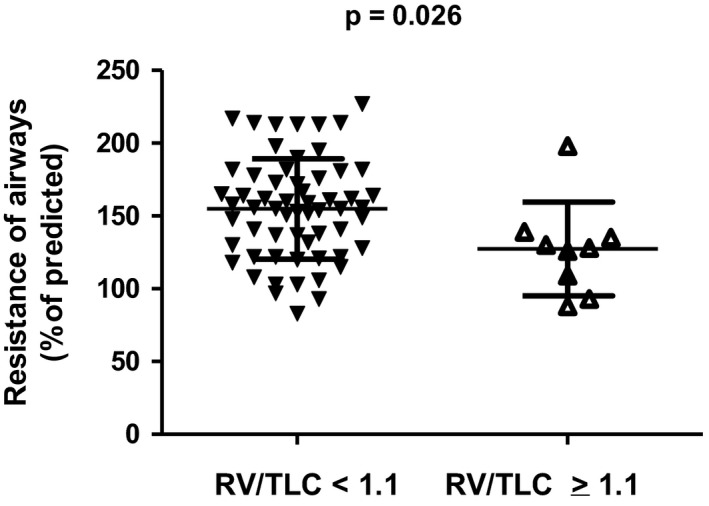
Resistance of airways below and above break point 5. Mean and standard deviation are given

**FIGURE 5:**
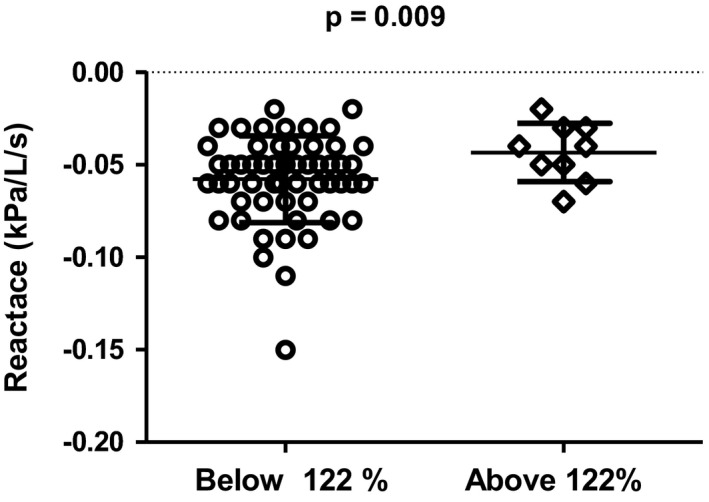
Oscillometric reactance below and above break point 2. Mean and standard deviation are given

As we analyzed lung function below and above some of the break points, there was variation in the results, probably depending on the small numbers between the break points. However, there were some findings for at least two of them (see Table 5 and Figures 3, 4, 5). First of all, as we compared the subjects below break points 2 and 5 (*N* = 58) and above them (*N* = 9), the subjects were almost similar according to age and BMI. FEV1/VC and FEV1/FVC were lower above break point 2 than below break point 2 (see Figure 3) or break point 5. However, the resistance of airways (Rtot%) was lower above them than it was below them (see Figure 4; *p* = 0.018). Oscillometric reactance was significantly higher above break point 2 and break point 5 (see Figure 5).

**TABLE 5:** Anthropometric data and some lung function test results of subjects above and below break points 2 and 5

	Break point 2. Results related to the break point of Trapped air versus VC% (VC 122%)			Break point 5. Results related to the break point of Trapped air versus RV/TLC (trapped air 1.1 L)		
	Below break point *N* = 58	Above break point *N* = 9	*p*‐value	Below break point *N* = 58	Above break point *N* = 9	*p*‐value
Age (year)	34.1 (9.1)	35.4 (9.5)	0.675	34.2 (9.1)	35.2 (10.2)	0.767
Weight (kg)	82.5 (8.2)	84.7 (9.6)	0.439	82.2 (8.5)	86.6 (6.2)	0.146
Height (cm)	180.1 (5.2)	180.3 (5.6)	0.782	180 (85.3)	181 (4.6)	0.556
BMI (kg/m^2^)	25.4 (2.1)	26.0 (2.7)	0.640	25.3 (2.2)	26.4 (1.9)	0.172
FEV1/FVC %	0.94 (1.12)	0.70 (0.06)	**0.005**	0.77 (0.078)	0.75 (0.06)	**0.049**
FEV1/FVC (% of predicted)	95.9 (8.3)	86.0 (8.2)	**0.003**	93.7 (7.8)	90.0 (5.9)	0.085
FEV1/VC %	0.78 (0.07)	0.69 (0.08)	**0.001**	0.93 (1.18)	0.74 (0.04)	0.289
FEV1/VC (% of predicted)	94.5(7.09)	85.2 (6.8)	**0.002**	95.0 (9.07)	92.2 (7.9)	0.325
Rtot (kPa*s/L)	0.19 (0.05)	0.16 (0.03)	**0.030**	0.19 (0.05)	0.16 (0.05)	0.341
Rtot (%)	156.3 (34.9)	127.34 (23.4)	**0.015**	156.3 (33.9)	127.3 (32.1)	**0.026**
DLCO (mmol/min/kPa)	11.3 (1.4)	12.0 (1.85)	0.339	11.4 (1.4)	11.7 (1.9)	0.051
DLCO (%)	102.1 (12.2)	107.6 (11.8)	0.170	102.7 (11.9)	104.44 (14.9)	0.408
DLCO/VA (mmol/min/kPa/L)	1.6 (0.17)	1.4 (1.18)	**0.005**	1.58 (0.19)	1.51 (0.13)	0.154
DLCO/VA (%)	99.1 (10.3)	88 (9.8)	**0.005**	98.4 (11.0)	2.4 (8.7)	0.258
R5 (kPa/L/s)	0.24 (0.06)	0.24 (0.04)	0.588	0.241 (0.06)	0.246 (0.06)	0.978
R5%	93.7 (23.5)	90.3 (18.8)	0.919	93.1 (22.9)	94.8 (24.6)	0.917
X5 (kPa/L/s)	−0.059 (0.02)	−0.039 (0.01)	**0.009**	−0.058 (0.022)	−0.043 (0.02)	**0.024**
X5%	−310.1 (1201.4)	468.0 (1575.6)	**0.037**	−228.1 (1173.8)	−119.2 (662.7)	0.297

Bold values indicates *p*‐values < 0.05 are significant.

### 3.4 Multiple comparisons

Calculations to control multiple comparisons (corrected q‐values and false discovery rates) provided no change in the results or main findings of the study.

### 3.5 Gas compression

Gas compression at the MEF50 and MEF75 levels tended to be larger in the divers than in the controls, but the difference did not reach significance (see Table 2).

### 3.6 High‐resolution computed tomography (HRCT)

We performed a HRCT of the lungs for the diver (age 52) with one of the highest amounts of trapped air (1.51 L). The lungs presented with several sets of small emphysema bullae (maximal size: 6–7 mm).

## 4 DISCUSSION

The Finnish Navy divers had larger lungs than the non‐diving reference group. Furthermore, our results suggest that large lungs can present larger volumes of trapped air than can merely be explained by the size of the lungs. The amount of trapped air was higher in many of those individuals with large lungs than formerly reported in healthy subjects, and in some of them, the amount was close to the values observed in patients with pulmonary diseases (Schaaning & Gulsvik, 1973; Sovijärvi et al., 1980).

In our study, the relation of VC to trapped air was almost stable until the volume of about 7.31 L or 122% of the predicted value of VC, but at volumes higher than that, the volume of trapped air started to increase significantly. Interestingly, the increased volume of trapped air was also associated with increased RV and decreased ERV. This association only appeared with large volumes of trapped air as the relation of RV and RV/TLC to trapped air was almost stable until the volume of 1.1 L of trapped air. Thereafter, both RV and RV/TLC started to increase. Later, at the volume of 1.6 L of trapped air also ERV started to decrease. However, the increase in the volume of trapped air was not associated with other pulmonary pathology, including signs of obstruction, functional airway compression, or a reduction in diffusing capacity. Subjects above the break points had a lower FEV1/FVC relation, lower Rtot, and higher reactance, suggesting that obstruction does not explain the finding.

The etiology of increased RV can be multifactorial, for instance, it can involve emphysema bullae, pockets or spaces of gases, dilated bronchioles, or functional airway compression. Small bullae and lobular air trapping are also common in the healthy population, but they are more prevalent in smokers and older individuals. Previously, in a Dutch report (Hulst et al., 2011) the only common factor in divers with PBT was large lungs, and the divers also presented with HRCT findings, including air trapping, large bullae, and multiple blebs. However, the reported prevalence of lobular air trapping among healthy Dutch military divers is high making it difficult to prove a causative association. In our study, we were only able to perform a lung HRCT scan for the diver with one of the highest amounts of air trapping. The scan revealed a high number of small emphysema bullae. The high number of air‐containing cavities can explain the increased air trapping—at least in this individual.

Physiological airway compression can explain some level of air trapping as bronchioles collapse before exhalation is complete. In our study, the level of functional airway compression was not associated with air trapping. Pronounced functional airway compression would be disadvantageous to divers as it leads to air trapping, reduced ventilation, and eventually to CO_2_ retention. The retention of CO_2_ is aggravated by both physical exercise and the increased density of inhaled gas with increased diving depth (Mitchell et al., 2007). In divers with healthy lungs, physiological airway compression would probably not increase the risk of PBT of ascent. However, the opposite could be true for a diver with underlying lung diseases, for instance, emphysema, interstitial lung disease, or obesity (Piirilä et al., 2014).

As we analyzed trapped air related to several lung function tests, we found eight break points and found that the trapped air rapidly increased in some of the individuals with large lungs after a break point, although above some break points there were only a few individuals. We analyzed the two most representative break points. We found a suggestion of peripheral obstruction based on significantly lower FEV1/FVC above the break points. However, in further examinations the plethysmographic resistance of airways was lower in those above the break points than in those below the break points.

This could all suggest that there was no indication of obstruction present in those above the break points, but rather that it is an indication of changes in elastic properties with increased closing tendency, based on CV measurement. The development of increased closing tendency might be generated during the compression–decompression phenomena associated with diving. Changes in elastic properties of the lung tissue would lead to an increase in RV and a predisposition to both the formation of bullae and functional airway compression with air trapping. However, these findings need further study.

In the present study, there was no indication that the lowered findings of the last portion of the spirogram would represent a real obstructive pulmonary disease. This is congruent with the study from New Zealand stating that the changes in spirometric measurements reflect more age‐related changes than real obstruction (Sames et al., 2018). In our study, none of those with lowered FEV1/FVC, FEV1/VC, or MEF25‐50 showed a bronchodilation response. Also, the resistance of airways was normal in all the divers, and their closing capacity was even lower than in the controls. In addition, the diffusing capacity was normal in all of the divers and tended to be even higher than in the controls. Interestingly, the oscillometric reactance percentage of the reference values was higher in the divers than in the controls, which could suggest that the mechanism for higher lung volumes could be associated with the elastic properties of the lungs and an increase in the compliance of lung tissue, as stated earlier (Bickel et al., 2014; Elshout et al., 1994), although compliance measurements were not available here.

The strengths of the study are that the participants were nonsmoking, normal weight subjects with the mean age of about 40 years old, and they were studied with a large amount of lung function tests that were carefully performed. A limitation is that the diver population and especially the number of control subjects could have been larger. Another limitation is that pulmonary compliance was not measured and the conclusions on the involvement of compliance changes were made indirectly, mainly by oscillometric results.

The increased air trapping phenomenon with large lungs can be disadvantageous for divers or even a safety concern. Despite the previously mentioned report suggested an increase in PBT in military divers with large lungs (Hulst et al., 2011; Mets et al., 2012) and the fact that we have divers with large lungs in the navy, we have not had any PBT cases in the Finnish Navy. This may result from strict safety regulations in diving operations and health surveillance programs. The causality between large lungs and PBT is not well proven either.

In conclusion, the navy divers had larger lung volumes than the controls, and the largest lung volumes were associated with a disproportionally increased RV and spirometric results, suggesting bronchial obstruction. However, no obstruction was found in the divers, the results suggesting that changes in the elastic properties of lung tissue might have developed under repeated pressure variations during diving. Increased volumes of trapped air and RV may also indicate pulmonary pathology. However, the mechanisms need further study, but the increased volume of slowly ventilated air may remain disadvantageous for divers.

## CONFLICT OF INTERESTS

The authors declare that no conflict of interest exists with this submission.

## AUTHOR CONTRIBUTIONS

T.W., J.H., J.T., K.P., and P.P. conceived and designed the research; T.W. and P.P. performed the experiments; T.W., J.H., J.T., and P.P. analyzed the data; T.W., J.H., J.T., K.P., and P.P. interpreted the results of the experiments; J.H. prepared the figures; T.W. and P.P drafted the manuscript; T.W., J.H., J.T., K.P., and P.P. edited and revised the manuscript.
